# Whole-Genome Sequencing of Shiga Toxin–Producing *Escherichia coli* OX18 from a Fatal Hemolytic Uremic Syndrome Case

**DOI:** 10.3201/eid2705.204162

**Published:** 2021-05

**Authors:** Kenichi Lee, Atsushi Iguchi, Kazuhiro Uda, Sohshi Matsumura, Isao Miyairi, Kenji Ishikura, Makoto Ohnishi, Junji Seto, Kanako Ishikawa, Noriko Konishi, Hiromi Obata, Ichiro Furukawa, Hiromi Nagaoka, Hirotaka Morinushi, Natsuki Hama, Ryohei Nomoto, Hiroshi Nakajima, Hideaki Kariya, Mitsuhiro Hamasaki, Sunao Iyoda

**Affiliations:** National Institute of Infectious Diseases, Tokyo, Japan (K. Lee, M. Ohnishi, S. Iyoda);; University of Miyazaki, Miyazaki, Japan (A. Iguchi);; National Center for Child Health and Development, Tokyo (K. Uda, S. Matsumura, I. Miyairi, K. Ishikura);; Tokyo Metropolitan Children’s Medical Center, Tokyo (K. Uda);; Kanagawa Children’s Medical Center, Kanagawa, Japan (S. Matsumura);; Kitasato University School of Medicine, Tokyo (K. Ishikura);; Yamagata Prefectural Institute of Public Health, Yamagata, Japan (J. Seto);; Ibaraki Prefectural Institute of Public Health, Ibaraki, Japan (K. Ishikawa);; Tokyo Metropolitan Institute of Public Health, Tokyo (N. Konishi, H. Obata);; Kanagawa Prefectural Institute of Public Health, Kanagawa (I. Furukawa);; Shizuoka Institute of Environment and Hygiene, Shizuoka, Japan (H. Nagaoka, H. Morinushi);; Kobe Institute of Health, Hyogo, Japan (N. Hama, R. Nomoto);; Okayama Prefectural Institute for Environmental Science and Public Health, Okayama, Japan (H. Nakajima, H. Kariya);; Fukuoka Institute of Health and Environmental Sciences, Fukuoka, Japan (M. Hamasaki)

**Keywords:** Bacteria, E. coli, Og-typing, OX18, Shiga toxin–producing Escherichia coli, foodborne pathogens, STEC, whole-genome sequencing, Japan, food safety, enteric infections

## Abstract

We report a fatal case of hemolytic uremic syndrome with urinary tract infection in Japan caused by Shiga toxin–producing *Escherichia coli*. We genotypically identified the isolate as OX18:H2. Whole-genome sequencing revealed 3 potentially pathogenic lineages (OX18:H2, H19, and H34) that have been continuously isolated in Japan.

Shiga toxin–producing *Escherichia coli* (STEC) is a consequential foodborne pathogen worldwide. The most prevalent STEC O serogroups—O157, O26, O111, O103, O121, O145, and O45—cause severe symptoms, including bloody diarrhea and hemolytic uremic syndrome (HUS). These STECs usually carry the locus of enterocyte effacement (LEE) region, which is required for intimate bacterial adherence to host epithelial cells (*1*). However, LEE-negative STEC serotypes, including O104:H4 and O113:H21, can also cause outbreaks or severe cases (*2*,*3*). Although most severe cases develop from intestinal tract infections, HUS cases related to urinary tract infections have been reported (*4*). We report a fatal case of HUS in Japan caused by a LEE-negative strain identified as OX18:H2.

## The Case

In 2017, an 8-year-old girl in Japan was hospitalized for a urinary tract *E. coli* infection, which was treated with ceftazidime. Two days after hospitalization, she became unconscious. Laboratory results revealed anemia (hemoglobin 10.5 g/dL) with schistocytes; low platelet count (3.8 × 10^4^/µL); and elevated creatinine (1.38 mg/dL), total bilirubin (1.7 mg/dL), and lactate dehydrogenase (1,848 U/L). Magnetic resonance imaging of her head showed hyperintensity in the basal ganglia and thalamus, suggesting edema and necrosis. From the urine sample, we isolated a LEE-negative STEC (strain JNE170426) carrying the Shiga toxin 2 gene (*stx2*). On the basis of these findings we diagnosed her condition as HUS with urinary tract infection. We performed intravenous high-dose methylprednisolone therapy, plasma exchange, and hemodialysis for HUS encephalopathy and renal failure, but after 12 days of intensive therapy, she died of HUS encephalopathy.

The isolated STEC did not show agglutination against commercial O1–O188 antisera (Denka Company Ltd., https://www.denka.co.jp; Statens Serum Institut, https://en.ssi.dk). However, comprehensive PCR-based O serogrouping (*5*) revealed that the isolate was classified into OX18, an atypical O serogroup originally identified from a nonpathogenic *E. coli* strain from a healthy sow (*6*). Using OX18-specific PCR screening of O-untypeable STEC isolates obtained during 2007–2019 by the National Institute of Infectious Diseases in Japan, we found 25 additional STEC OX18 isolates (Table). To characterize these isolates, we performed whole-genome sequencing (WGS) using MiSeq (Illumina, https://www.illumina.com). WGS results were analyzed as described elsewhere (*7*,*8*) with slight modification. We used BactSNP version 1.0.2 (http://platanus.bio.titech.ac.jp/bactsnp) (*9*) and Gubbins version 2.4.1 (https://sanger-pathogens.github.io) (*10*) for core genome SNP extraction. Public database strains used for the phylogenetic analysis are shown in Appendix Table 1. We deposited draft genome sequences and short-read sequencing data into the DDBJ/National Center for Biotechnology Information/European Nucleotide Archive database (BioProject accession no. PRJDB10421; Sequence Read Archive accession no. DRA010812).

**Table:** OX18 isolates used in study of whole-genome sequencing of Shiga toxin–producing *Escherichia coli* OX18 from a fatal hemolytic uremic syndrome case, Japan*

Strain	Year isolated	Source	Symptoms	H genotype	Phylogenetic group	MLST	*stx* subtype			Accession no.	
							*stx1*	*stx2*		Draft genome	Short reads
JNE101081	2010	Human	BD	H34	E	9185	1a	ND		BNCS00000000	SAMD00244533
JNE130471	NA	Swine	NA	H34	E	9185	1a	ND		BNCT00000000	SAMD00244534
JNE130573	2012	Human	D	H19	B1	205	ND	2a		BNCU00000000	SAMD00244535
JNE133347	2012	Human	AC	H2	B1	9397	ND	2e		BNCV00000000	SAMD00244536
JNE150598	2015	Human	BD	H19	B1	205	ND	2a		BNCW00000000	SAMD00244537
JNE151350	2015	Human	AC	H19	B1	205	ND	2d		BNCX00000000	SAMD00244538
JNE170426	2017	Human	HUS, death	H2	B1	847	ND	2a		BNCY00000000	SAMD00244539
JNE180342	2018	Human	AC	H8	B1	Novel	1a	2d		BNCZ00000000	SAMD00244540
JNE181771	2018	Human	HUS	H19	B1	205	ND	2a		BNDA00000000	SAMD00244541
JNE182474	2018	Human	BD	H19	B1	205	ND	2a		BNDB00000000	SAMD00244542
JNE182523	NA	Human	NA	H19	B1	205	ND	2a		BNDC00000000	SAMD00244543
JNE191031	2019	Human	BD	H19	B1	205	ND	2a		BNDD00000000	SAMD00244544
JNE192124	2019	Human	AC	H19	B1	205	ND	2a		BNDE00000000	SAMD00244545
JNE192333	2019	Human	AC	H28	B1	1056	1d	ND		BNDF00000000	SAMD00244546
A140161	2010	Cattle	NA	H19	B1	205	ND	2a		BNDG00000000	SAMD00244547
A140164	2010	Cattle	NA	H19	B1	205	ND	2a		BNDH00000000	SAMD00244548
A140165	2010	Cattle	NA	H19	B1	205	ND	2a		BNDI00000000	SAMD00244549
A140286	2012	Cattle	NA	H19	B1	205	ND	2a		BNDJ00000000	SAMD00244550
A140453	2010	Cattle	NA	H19	B1	Novel	1a	2a		BNDK00000000	SAMD00244551
A140462	2010	Cattle	NA	H19	B1	205	1a	ND		BNDL00000000	SAMD00244552
A140486	2014	Cattle	NA	H19	B1	205	ND	2a×2†		BNDM00000000	SAMD00244553
A150011	2014	Cattle	NA	H19	B1	205	ND	2a×2†		BNDN00000000	SAMD00244554
A150026	2014	Cattle	NA	H19	B1	205	ND	2a×2†		BNDO00000000	SAMD00244555
A150037	2015	Cattle	NA	H19	B1	205	ND	2a×2†		BNDP00000000	SAMD00244556
A150038	2015	Cattle	NA	H19	B1	205	ND	2a×2†		BNDQ00000000	SAMD00244557
A150039	2015	Cattle	NA	H19	B1	205	ND	2a×2†		BNDR00000000	SAMD00244558

*AC, asymptomatic carrier; BD, bloody diarrhea; D, diarrhea; HUS, hemolytic uremic syndrome; NA, not available; ND, not detected.  †2a×2, two copies of *stx2a* were detected.

In silico analysis revealed that none of the STEC OX18 isolates carried LEE; we classified them into 5 H-genotypes: H2 (n = 2), H8 (n = 1), H19 (n = 20), H28 (n = 1), and H34 (n = 2) (Table). Core-genome SNP phylogeny revealed that OX18 isolates with the same H-types formed closely related groups (Table; Figure). Isolates from patients belonged to OX18:H2, H19, and H34; isolates belonging to OX18:H8 and H28 were obtained from asymptomatic carriers. The OX18 isolate from the case-patient who died of HUS (strain JNE170426) belonged to H2 and carried *stx2a* and several virulence genes, including STEC autoagglutination adhesin (*saa*), subtilase toxin (*sub*), enterohemolysin (*ehx*), and serine protease (*espP*) (Appendix Table 2). These regions showed high similarity (>99%) to a large plasmid of STEC O104:H21 strain CFSAN002236 (*11*). Therefore, these virulence factors are likely to be encoded in similar plasmids.

**Figure:**
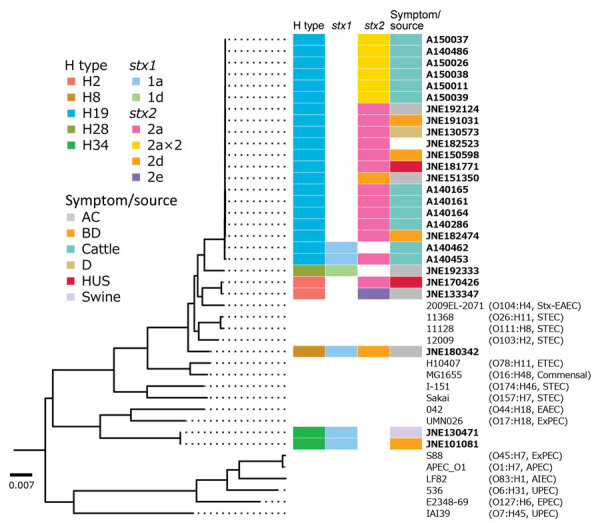
Maximum-likelihood phylogeny of STEC OX18 from a patient in Japan and other *Escherichia coli* strains. Isolate identifications of STEC OX18 are shown in bold. Colored boxes indicate collection countries, stx profiles, and symptoms of human carrier or source of the STEC OX18 isolates, as shown in the keys. Serotype and pathotype information of non-OX18 *E. coli* strains are shown in parentheses. The tree was rooted by *E. fergusonii* ATCC35469. AC, asymptomatic carrier; BD, bloody diarrhea; D, diarrhea; HUS, hemolytic uremic syndrome; STEC, Shiga toxin–producing *E. coli*; Stx, Shiga toxin. APEC, avian pathogenic *E. coli*; AIEC, adherent/invasive E. coli; EAEC, enteroaggregative *E. coli*; EPEC, enteropathogenic *E.coli*; ExPEC, extraintestinal pathogenic *E. coli*; UPEC, uropathogenic *E. coli.* Scale bar indicates number of substitutions per site.

On the other hand, the other OX18:H2 isolate (strain JNE133347) from an asymptomatic carrier did not carry the virulence genes described above but carried genes for Shiga toxin 2e (*stx2e*), heat-stable enterotoxin (*st*), and Pap fimbriae (*pap*). Of note, the other isolates obtained from HUS belonged to OX18:H19 and were phylogenetically close to OX18:H2 (Figure). The OX18:H19 lineage showed a similar virulence profile to the OX18:H2 isolate from the fatal HUS case, and carried *saa*, *sub*, *ehx*, and *espP* virulence genes on plasmid-like elements. All bovine isolates in our study were grouped into this serotype. OX18:H19 isolates from humans and bovines could not be distinguished by their lineages, suggesting that cattle can be a reservoir for that lineage. We identified OX18:H34 in isolates that carried *pap* as an adhesin from a patient with bloody diarrhea and from swine. The other isolates, from asymptomatic carriers, we classified into H8 and H28. The OX18:H8 isolate carried *saa*, *sub*, *ehx*, and *espP* on plasmid-like elements, similar to the H19 lineage. Meanwhile, the OX18:H28 isolate did not carry adherence factors known in pathogenic *E. coli*, including LEE genes, *saa*, *pap*, *aggR*, *afaD*, F4, F6, F17, F18, or F41.

Among LEE-negative STEC isolates, *saa*-positive STEC has often been reported in patients with severe symptoms (*2*,*3*). WGS analyses of *saa*-positive STEC O104:H21 and O113:H21 revealed that they carry a large plasmid (>100 kb) with several virulence genes, including *saa* and *sub*. Because the draft genomes of *saa*-positive OX18:H2 and H19 showed high similarity to the plasmid, it is plausible that they carry a similar large plasmid. The source or natural reservoir of these lineages was unclear. However, some OX18:H19 isolates have been obtained from cattle, suggesting that cattle or fecally contaminated foods can be a source of the infection. In addition to these lineages, OX18:H34 was found to cause severe symptoms in humans. We were unable to elucidate the pathogenesis and natural reservoir of the lineage because of the small sample size of our study; further studies are required.

## Conclusion

In this study, we report a HUS case with urinary tract infection caused by a STEC belonging to the emerging O serogroup OX18. Our retrospective survey revealed that the novel pathogenic STECs OX18:H2, H19, and H34 have been continually isolated from humans and cattle. However, commercial antisera cannot identify these lineages. Elucidating the transmission routes and natural reservoirs of the bacteria is essential to control infection. DNA-based serotyping methods, including Og/Hg typing (*6*,*12*,*13*) and whole-genome sequencing (*7*,*14*,*15*), would be helpful for identification and surveillance of these potentially pathogenic lineages.

Appendix Additional information on *Escherichia* strains and virulence genes in study of whole-genome sequencing of Shiga toxin–producing *E. coli* OX18 from a fatal hemolytic uremic syndrome case, Japan.
