# Supplementary material for: Whole-Genome Sequencing of Shiga Toxin–Producing Escherichia coli OX18 from a Fatal Hemolytic Uremic Syndrome Case
Source: Emerg Infect Dis. 2021 May;27(5):1509–12. doi: 10.3201/eid2705.204162 (PMC8084502; doi:10.3201/eid2705.204162)
Supplement: Appendix — Additional information on Escherichia strains and virulence genes in study of whole-genome sequencing of Shiga toxin–producing E. coli OX18 from a fatal hemolytic uremic syndrome case, Japan. [file 20-4162-Techapp-s1.pdf]

Article DOI: https://doi.org/10.3201/eid2705.204162

Whole-Genome Sequencing of Shiga Toxin–Producing *Escherichia coli* OX18 from a Fatal Hemolytic Uremic Syndrome Case

Appendix

Appendix Table 1. *Escherichia* strains used for phylogenetic analysis

Isolate ID	Serotype or species	Pathotype	Phylogenetic group	Accession no.	Source	Year	Place
APEC_O1	O1:H7	APEC*	B2	CP000468	Poultry	†	USA
12009	O103:H2	STEC	B1	AP010958	Human	2001	Japan
2009EL-2071	O104:H4	*Stx*-EAEC	B1	CP003301	Human	2009	Georgia
11128	O111:H8	STEC	B1	AP010960	Human	2001	Japan
E2348/69	O127:H6	EPEC	B2	FM180568	Human	1969	UK
Sakai	O157:H7	STEC	E	BA000007	Human	1996	Japan
MG1655	O16:H48	Commensal	A	CP025268	†	†	USA
UMN026	O17:H18	ExPEC	D	CU928163	Human	1999	USA
I-151	O174:H46	STEC	E	SAMN02732277	Human	2005	Germany
11368	O26:H11	STEC	B1	AP010953	Human	2001	Japan
042	O44:H18	EAEC	D	FN554766	Human	1983	Peru
S88	O45:H7	ExPEC	B2	CU928161	Human	1999	France
536	O6:H31	UPEC	B2	CP000247	Human	1982	Germany
IAI39	O7:H45	UPEC	F	CU928164	Human	1980s	France
H10407	O78:H11	ETEC	A	FN649414	Human	1973	Bangladesh
LF82	O83:H1	AIEC	B2	CU651637	Human	†	France
ATCC35469	*E. fergusonii*	NA	NA	CU928158	Human	†	USA

*AIEC, adherent invasive *E. coli*; APEC, avian pathogenic *E. coli*; EAEC, enteroaggregative *E. coli*; EPEC, enteropathogenic *E. coli*; ETEC, enterotoxigenic *E. coli*; ExPEC, extraintestinal pathogenic *E. coli*; NA, not applicable; STEC, Shiga toxin-producing *E. coli*; Stx-EAEC, Shiga toxin-producing enteroaggregative *E. coli*; UPEC, uropathogenic *E. coli*.
†Data not available.

Appendix Table 2. Virulence gene profile detected by VirulenceFinder 2.0 (https://cge.cbs.dtu.dk/services/VirulenceFinder/).

	H		Virulence genes																																					
Strain	type	MLST	*air*	*astA*	*cba*	*cea*	*celb*	*chuA*	*cia*	*cib*	*cma*	*cvaC*	*ehxA*	*eilA*	*epeA*	*espI*	*espP*	*gad*	*hlyA*	*hra*	*iha*	*ireA*	*iroN*	*iss*	*katP*	*lpfA*	*mchF*	*ompT*	*papA_F19*	*papA_F20*	*papC*	*sepA*	*sta1*	*stb*	*stx1*	*stx2*	*subA*	*terC*	*traT*	*usp*
JNE101081	H34	9185	1‡	0	0	0	0	1	1	0	0	1	0	1	0	0	0	1	0	0	0	1	1	1	1	0	1	1	0	1	1	0	0	0	1	0	0	1	0	1
JNE130471	H34	9185	1	0	0	0	0	1	1	0	0	1	0	1	0	0	0	1	0	0	0	1	1	1	1	0	1	1	0	1	1	0	0	0	1	0	0	1	0	1
JNE130573	H19	205	0	0	0	0	0	0	1	0	0	0	1	0	0	0	1	1	0	0	1	0	0	1	0	1	0	1	0	0	0	0	0	0	0	1	1	1	1	0
JNE133347	H2	9397	0	0	1	0	0	0	0	0	0	0	0	0	0	0	0	1	0	1	0	0	0	1	0	1	0	1	1	0	0	1	1	1	0	1	0	1	1	0
JNE150598	H19	205	0	0	0	0	0	0	0	0	0	0	0	0	0	0	0	1	0	0	0	0	0	1	0	1	0	1	0	0	0	0	0	0	0	1	0	1	0	0
JNE151350	H19	205	0	0	0	0	0	0	1	0	0	0	1	0	0	0	1	1	0	0	1	0	0	1	0	1	0	1	0	0	0	0	0	0	0	1	1	1	1	0
JNE170426	H2	847	0	0	0	0	1	0	1	1	0	0	1	0	1	0	1	1	0	0	1	0	0	1	0	1	0	1	0	0	1	0	0	0	0	1	1	1	1	0
JNE180342	H8	Novel*	0	0	1	1	0	0	1	0	0	1	1	0	0	0	1	1	0	1	1	0	0	1	0	1	1	1	0	0	0	0	0	0	1	1	1	1	1	0
JNE181771	H19	205	0	0	1	0	0	0	1	0	1	0	1	0	0	0	1	1	0	0	1	0	0	1	0	1	0	1	0	0	0	0	0	0	0	1	1	1	1	0
JNE182474	H19	205	0	0	0	1	0	0	1	1	0	0	1	0	1	0	1	1	0	0	1	0	0	1	0	1	0	1	0	0	0	0	0	0	0	1	1	1	1	0
JNE182523	H19	205	0	0	0	0	0	0	1	0	0	0	1	0	0	0	1	1	0	0	1	0	0	1	0	1	0	1	0	0	0	0	0	0	0	1	1	1	1	0
JNE191031	H19	205	0	0	0	0	0	0	1	0	0	0	1	0	0	0	1	1	0	0	1	0	0	1	0	1	0	1	0	0	0	0	0	0	0	1	1	1	1	0
JNE192124	H19	205	0	0	0	1	0	0	1	0	0	0	1	0	0	0	1	1	0	0	1	0	0	1	0	1	0	1	0	0	0	0	0	0	0	1	1	1	1	0
JNE192333	H28	1056	0	1	0	0	0	0	1	0	0	0	0	0	0	0	0	1	1	1	0	0	0	1	0	1	0	1	0	0	0	0	0	0	1	0	0	1	1	0
A140161	H19	205	0	0	0	1	0	0	1	1	0	0	1	0	1	0	1	0	0	0	1	0	0	1	0	1	0	1	0	0	0	0	0	0	0	1	1	1	1	0
A140164	H19	205	0	0	0	1	0	0	1	1	0	0	1	0	1	0	1	0	0	0	1	0	0	1	0	1	0	1	0	0	0	0	0	0	0	1	1	1	1	0
A140165	H19	205	0	0	0	1	0	0	1	1	0	0	1	0	1	0	1	1	0	0	1	0	0	1	0	1	0	1	0	0	0	0	0	0	0	1	1	1	1	0
A140286	H19	205	0	0	0	1	1	0	1	1	0	0	1	0	1	0	1	1	0	0	1	0	0	1	0	1	0	1	0	0	0	0	0	0	0	0	1	1	1	0
A140453	H19	Novel†	0	0	0	1	0	0	1	0	0	1	1	0	0	1	0	1	0	1	1	0	0	1	0	1	0	1	0	0	0	0	0	0	1	1	0	1	1	0
A140462	H19	205	0	0	0	0	0	0	0	0	0	0	1	0	0	0	1	1	0	0	1	0	0	1	0	1	0	1	0	0	0	0	0	0	1	0	0	1	1	0
A140486	H19	205	0	0	0	0	0	0	1	0	0	0	1	0	0	0	1	1	0	0	1	0	0	1	0	1	0	1	0	0	0	0	0	0	0	1	1	1	1	0
A150011	H19	205	0	0	0	0	0	0	1	0	0	0	1	0	0	0	1	1	0	0	1	0	0	1	0	1	0	1	0	0	0	0	0	0	0	1	1	1	1	0
A150026	H19	205	0	0	0	0	0	0	1	0	0	0	1	0	0	0	1	1	0	0	1	0	0	1	0	1	0	1	0	0	0	0	0	0	0	1	1	1	1	0
A150037	H19	205	0	0	0	0	0	0	1	0	0	0	1	0	0	0	1	1	0	0	1	0	0	1	0	1	0	1	0	0	0	0	0	0	0	1	1	1	1	0
A150038	H19	205	0	0	0	0	0	0	1	0	0	0	1	0	0	0	1	0	0	0	1	0	0	1	0	1	0	1	0	0	0	0	0	0	0	1	1	1	1	0
A150039	H19	205	0	0	0	0	0	0	1	0	0	0	1	0	0	0	1	1	0	0	1	0	0	1	0	1	0	1	0	0	0	0	0	0	0	1	1	1	1	0

*Same profile to ST5978, except one point mutation in *adk.*
†Same profile to ST205, except one point mutation in *gyrB.*
‡1, presence; 0, absence.
